# Practical based approach to left main bifurcation stenting

**DOI:** 10.1186/s12872-016-0227-1

**Published:** 2016-02-19

**Authors:** Jung-Min Ahn, Pil Hyung Lee, Seung-Jung Park

**Affiliations:** Department of Cardiology, University of Ulsan College of Medicine, Asan Medical Center, 388-1 Poongnap-dong, Songpa-gu, Seoul 138-736 Korea

**Keywords:** Left main coronary artery, Percutaneous coronary intervention, Bifurcation, Intravascular ultrasound, Fractional flow reserve

## Abstract

Despite the recent developments that have been made in the field of percutaneous left main (LM) intervention, the treatment of distal LM bifurcation remains challenging. The provisional one-stent approach for LM bifurcation has shown more favorable outcomes than the two-stent technique, making the former the preferred strategy in most types of LM bifurcation stenosis. However, elective two-stent techniques, none of which has been proven superior to the others, are still used in patients with severely diseased large side branches to avoid acute hemodynamic compromise. Selecting the proper bifurcation treatment strategy using meticulous intravascular ultrasound evaluation for side branch ostium is crucial for reducing the risk of side branch occlusion and for improving patient outcomes. In addition, unnecessary complex intervention can be avoided by measuring fractional flow reserve in angiographically isolated side branches. Most importantly, good long-term clinical outcomes are more related to the successful procedure itself than to the type of stenting technique, emphasizing the greater importance of optimizing the chosen technique than the choice of method.

## Background

Results of randomized trials and observational studies found that percutaneous coronary intervention (PCI) is a potential alternative to bypass surgery for patients with unprotected left main (LM) coronary artery stenosis [1]. However, PCI for LM bifurcation is technically demanding and has been associated with high rates of adverse clinical events [2]. In addition, a lack of randomized clinical trials focusing on distal LM intervention has often led to uncertainties regarding the optimal stenting strategy. In general, based on non-randomized studies and extrapolations from the results of non-LM bifurcation trials, the provisional one-stent approach has been considered as a preferred strategy over the elective two-stent technique for patients with LM bifurcation disease. In practice, however, two-stent techniques are chosen more frequently for LM bifurcation than for non-LM lesions due to concerns regarding the ischemic myocardial volume, which would be jeopardized by adverse events [3]. This review therefore discusses the optimal methods of distal LM bifurcation stenting from a practical point of view.

### Outcomes of provisional one-stent and two-stent technique

**Fig. 1:**
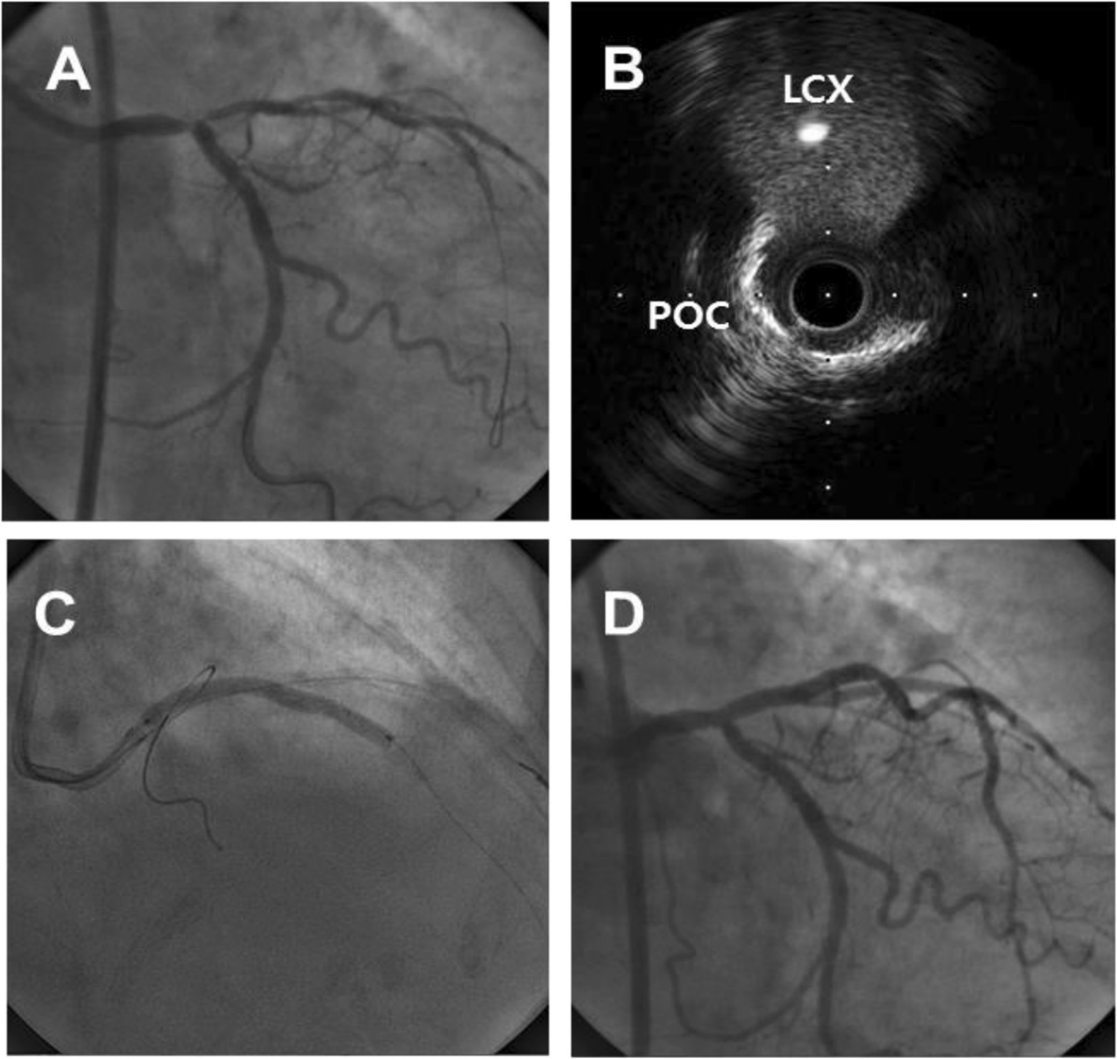
Provisional approach for distal left main stenosis. Coronary angiography showed a “true” LM bifurcation lesion (Medina 1.1.1) (**a**) while intravascular ultrasound revealed very minimal disease at the ostium of the left circumflex artery (**b**). Provisional single stenting was performed (**c**), with the final angiogram showing an acceptable result without side branch compromise (**d**)

The provisional approach is a single-stent strategy that allows the positioning of a second stent, if required (Fig. 1). Similar to non-LM bifurcations, several studies reported that, compared with two-stent techniques, the provisional one-stent approach for distal LM bifurcation was associated with more favorable outcomes, including lower risks of major adverse cardiac events [4–6], death [6], myocardial infarction [5, 6], and target vessel revascularization [5–7] (Table 1). In addition, the provisional one-stent approach was found to reduce the risk of stent thrombosis [6, 7]. Based on these results, the provisional one-stent approach has been preferred in the treatment of LM bifurcation stenosis [8], with more than 60 % of patients with LM bifurcation in real-world practice treated using the provisional one-stent technique [5]. However, all previous studies were observational, suggesting the need for randomized controlled studies to properly evaluate the superiority of the provisional approach over double stenting in patients with LM bifurcation disease.

**Table 1:** Outcomes of provisional single stenting versus double stenting

Reference	Year	Numbers of patients		FU (M)	Adjusted hazard ratio (95 % confidence interval)^a^				
		Provisional approach	Double stenting		MACE	Death or MI	Death	MI	TVR
Palmerini [4]	2008	456	317	24	0.48 (0.33–0.69) *p* = 0.001	0.38 (0.17–0.85) *p* = 0.018	-	-	-
Toyofuku [7]	2009	261	119	36	-	-	0.61 (0.34–1.08) *p* = 0.09	-	0.32 (0.18–1.21) *p* < 0.01
Kim [5]	2011	234	158	36	0.89 (0.22–0.67) *p* < 0.001	-	0.77 (0.28–2.13) *p* = 0.62	0.38 (0.19–0.78) *p* = 0.008	0.16 (0.05–0.57) *p* = 0.005
Song [6]	2014	509	344	36	0.42 (0.28–0.63) *p* < 0.001	0.48 (0.25–0.93) *p* = 0.03	0.30 (0.11–0.81) *p* = 0.02	0.41 (0.18–0.95) *p* = 0.04	0.47 (0.32–0.69) *p* < 0.01

*Abbreviations*: *FU* follow-up, *M* months, *MACE* major adverse cardiac events, *MI* myocardial infarction, *TVR* target vessel revascularization

^a^Hazard ratios are for patients undergoing the provisional approach, compared with patients undergoing double stenting

### Selecting a left main bifurcation treatment strategy

**Fig. 2:**
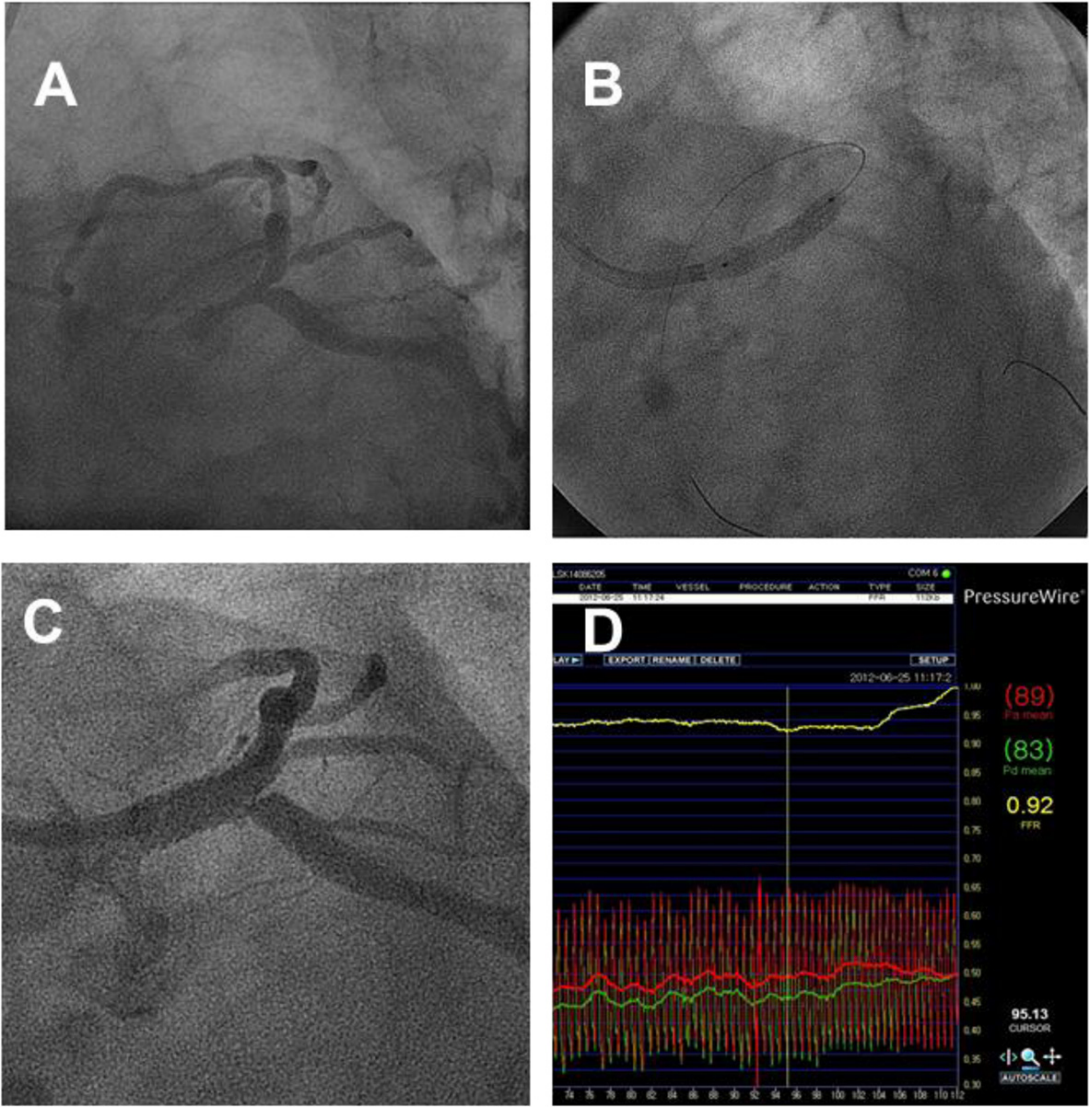
Fractional flow measurement after main vessel stenting. A patient with a distal LM bifurcation disease (**a**) underwent provisional one-stent implantation (**b**). After main vessel stenting, significant stenosis was observed at the ostium of the left circumflex artery (**c**). However, fractional flow reserve value was 0.92, indicating functionally insignificant stenosis (**d**), and suggesting that additional procedures were unnecessary

Because of the large myocardial volume supplied by the left circumflex artery (LCX) in many patients, the possibility of circulatory collapse after main vessel (MV) stenting should always be considered. Therefore, the presence or absence of significant disease in the ostium of the LCX is regarded as an important factor in choosing a stenting strategy. The provisional one-stent approach is preferred for LM bifurcations with insignificant stenosis at the ostial LCX or a non-dominant left coronary system (Fig. 2). By contrast, the elective two-stent technique is preferred in patients with significant ostial stenosis of the LCX with a dominant left coronary arterial system [9, 10]. Fractional flow reserve (FFR) evaluation for the side branch (SB) has provided valuable information on the relation between physiological and angiographic severity and can be useful to make correct choice of the treatment strategy [11]. Table 2 summarizes the selection criteria for stenting strategies based on the anatomic features involving the LM bifurcation.

**Table 2:** Selection criteria for the provisional one-stent approach versus the planned two-stent technique

Strategy	Anatomical features
Favors the Provisional Approach	• Insignificant stenosis at the ostial LCX with MEDINA classification 1,1,0 or 1,0,0 • Small LCX <2.5 mm in diameter • Diminutive LCX, right dominant coronary system • Wide angle between LAD and LCX • No concomitant disease or only focal disease in LCX
Favors the Two-Stent Technique	• Significant stenosis at the ostial LCX with MEDINA classification 1,1,1 or 1,0,1 or 0,1,1 • Large LCX ≥2.5 mm in diameter • Diseased left dominant coronary system • Narrow angle between LAD and LCX • Concomitant diffuse disease in LCX

*Abbreviations*: *LAD* left anterior descending artery, *LCX* left circumflex artery

In general, LM bifurcation disease is mostly diffuse, not focal [12], and angiography is inaccurate in assessing the disease severity of both branch ostia [13]. Thus angiography-guided intervention may lead to SB occlusion for a “true” bifurcation or unnecessary complex intervention that may be preventable. As intravascular ultrasound (IVUS) provides more accurate information on the disease status of the distal LM complex, including the LCX ostium, pre-procedural IVUS evaluation is very helpful in selecting an appropriate and safe stenting strategy. Previous studies reported that the use of IVUS reduced the risk of SB occlusion after MV stenting in coronary bifurcation lesions [9]. Moreover, IVUS-guided PCI for LM disease has been associated with low mortality rates [14, 15]. In association with the functional concept, IVUS-derived minimal lumen >3.7 mm^2^ or plaque burden <56 % in the LCX ostium can exclude functional SB compromise (FFR <0.80) after MV stenting in treating LM bifurcations [16]. However, in addition to these absolute numerical values reflecting lumen patency, relative plaque distribution [17] and the presence of calcified plaque [18] should also be considered in order to avoid the SB compromise after MV stenting.

### Determining SB intervention using the provisional one-stent approach

Patients who develop ischemic symptoms or signs after MV stenting attributable to SB compromise definitely require further SB intervention. However, it remains unclear whether further treating asymptomatic angiographic stenosis of an SB ostium after MV stenting provides clinical advantages. Nevertheless, in practice, additional SB interventions are frequently performed, primarily because of concerns regarding the future likelihood of SB deterioration.

Two small pilot studies suggested the benefit of FFR-guided decision making for SB intervention after MV stenting in LM bifurcation [16, 19]. These studies reported a considerable discrepancy between angiographic stenosis and FFR values, in that less than one-third of angiographically isolated LCX ostia were functionally significant (FFR, <0.80). This finding, and results suggesting that a FFR >0.80 is a strong predictor of favorable survival and low event rates in patients with coronary artery disease, including intermediate LM disease [20, 21], indicates that incorporating the FFR-guided PCI strategy to treat the isolated LCX may reduce the incidence of additional SB intervention and associated procedure-related complications. However, long-term clinical trials are needed to validate this FFR-guided SB approach in LM bifurcation stenting.

### Elective two-stent techniques

A planned two-stent approach can be attempted if the operator is concerned about acute complications, including hemodynamic compromise and peri-procedural myocardial infarction following SB loss. Current two-stent techniques commonly used for distal LM bifurcations include crush and its variants, culotte, and simultaneous kissing stent technique. Because few studies have evaluated the comparative outcomes of these two-stent techniques, there are still no clear guidelines in selecting a particular technique relative to the specific anatomy of the LM bifurcation lesion. Thus, selecting a proper stenting technique should depend on each the patient’s clinical manifestations, LM bifurcation morphology (e.g. the diameter of the two branches, bifurcation angle, severity of the ostial SB stenosis, extent of the MV disease), and the operator’s preference. Also, the operator should make every effort to understand the advantages and disadvantages of the various two-stent techniques.

The crush technique is a modified version of the T or kissing stent technique, in which the main branch (MB) stent crushes the SB stent against the MB wall. The classic crush technique is performed by retracting the SB stent 4–5 mm into the MB lumen, followed by crushing by the MB stent. In contrast with the shortcomings of T-stenting, which may leave a small un-stented gap at the SB ostium, the crush technique provides complete lesion coverage for the SB ostium and can be applied to any anatomic variation of true LM bifurcation. By contrast, the formation of three layers of struts covering the SB ostium just after MB stenting can make the final kissing balloon inflation (FKI) laborious and may cause unsatisfactory results. Because of these limitations and the complex procedures involved, the mini-crush technique was developed as a variant of the classic crush method. The mini-crush technique involves minimal (usually 1–2 mm) retraction of the SB stent into the MB before crushing, thus avoiding a large area with three strut layers and minimizing residual metallic stenosis at the SB ostium [22]. The double-kissing crush technique, another variant of the classic crush method, includes additional kissing balloon inflation between SB crushing and MV stenting and can further enhance stent apposition and facilitate FKI [23]. The culotte technique consists of the sequential implantation of two stents into both branches, with the MV stent implanted through the SB stent and protruding into the MB lumen. Consequently, the proximal MV is covered by two overlapping stents. This technique is suitable for all angles of bifurcations and provides near-perfect coverage of the SB ostium. However, it may cause intra-procedural acute closure of the MB after SB stenting, which can be catastrophic during interventions for distal LM disease. Since the proximal double stent layers can lead to delayed endothelialization and subsequent stent thrombosis, the stents should be overlapped minimally in the proximal MV segment whenever possible. Finally, the distal MB stent at the ostial left anterior descending artery can be under-expanded because of the positioning through the SB stent strut. The simultaneous kissing stent technique consists of the delivery and implantation of two stents, together with a two-barrel metallic carina, in the LM. The main advantage of this technique is that it guarantees the patency of both branches during the procedure and does not require rewiring for FKI. This technique is preferable in narrow-angle bifurcations, where the LM diameter is much larger than the diameters of the LAD and LCX. Unfortunately, this technique is now rarely used because of several concerns, including difficulty placing the stent proximal to the double barrel, the formation of a new diaphragmatic metal membrane, and difficulty in wiring in case of restenosis [24]. Nevertheless, its technical ease and rapidity make it an appropriate option for patients in highly unstable presentations, such as ST-elevation myocardial infarction involving the LM bifurcation. Importantly, one should always beware of the increased risk of bleeding since certain two stent techniques require 7–8Fr guiding catheter with femoral artery approach.

The particular characteristics of the LM bifurcation should be considered during intervention, in that the absolute difference between the reference vessel diameter of proximal MV and distal MB diameter is relatively large. In this regard, the proximal optimization technique may be of particular importance in LM bifurcation intervention [25]. This technique promotes adequate stent apposition in the LM stem, helps to avoid abluminal rewiring by a second wire, and facilitates rewiring the SB through a distal stent cell which is important for complete scaffolding of the SB ostium. Basically, the choice of stent diameter should be based on the distal MV reference diameter to minimize carina shifting [26, 27]. However, it is also important to select a stent where the platform in that particular nominal diameter accommodates expansion to the reference diameter of the LM coronary artery. The final decision should be made to balance these considerations.

Recently, several dedicated stents for bifurcations have been recently adopted for the treatment of LM disease [28]. These devices offer common advantages over conventional drug-eluting stents (DES) to cover the LM bifurcation segment. The design of these dedicated bifurcation stents and balloons conforms to the natural anatomy of the bifurcation and can facilitate a more effective scaffolding of the SB ostium. Furthermore, these devices provide easier access to the main and side branch which lowers the risk of SB loss during the procedure. Several studies have shown that stenting of LM with these new-dedicated stents is safe and effective both at short and mid-term follow-up [29–32]. Although, conceptually, the advantages offered by these devices may improve clinical outcomes after PCI of LM bifurcations, the success of currently available systems depends on specific anatomic features of bifurcations, and further studies are needed to define their role on LM bifurcations.

### Systematic kissing balloon inflation in provisional 1-stent approach

Systematic FKI after MV stenting is frequently performed in patients undergoing the provisional one-stent approach. Although FKI theoretically allows better strut contact together with the SB opening and is regarded as mandatory in performing any two-stent technique, its role in one-stent techniques remains unclear. A recent analysis involving 413 patients who underwent provisional single stenting at Asan Medical Center may provide insight into the use of FKI [33]. Of the 413 patients, 96 received FKI after MV stenting, whereas 318 did not. During a 2 year follow-up period, the rate of the composite of death, myocardial infarction, or target lesion revascularization did not differ significantly between these groups regardless of angiographic SB stenosis (12.5 % vs. 8.5 %; adjusted hazard ratio [HR], 1.10; 95 % confidence interval [CI], 0.49–2.49, *p* = 0.82). Moreover, although statistically insignificant, there was a trend toward more frequent target vessel revascularization in the FKI group (8.1 % vs. 4.8 %; adjusted HR, 1.12; 95 % CI, 0.40–3.11, *p* = 0.83). Another small observational study showed similar results [34]. Therefore, systematic FKI after MV stenting in the provisional one-stent strategy may not provide better long-term clinical outcomes and may be unnecessary.

### Importance of intravascular imaging-guided optimization

Stent under-expansion is the most important cause of DES failure. A minimal stent area (MSA) less than 5.0–5.5 mm2 was the best predictor on IVUS of first-generation DES restenosis or early thrombosis [35, 36]. However, there are no data suggesting the optimal MSA cutoff to predict restenosis and long-term clinical outcomes after DES treatment of LM stenosis—especially since in-stent restenosis can occur within any of the 4 segments: the LCX ostium, the LAD ostium, the polygon of confluence (POC), and the LM above the POC.

The optimal IVUS-MSA criteria for preventing in-stent restenosis were assessed in 403 patients undergoing DES implantation for LM coronary artery disease [37]. The best IVUS-MSA criteria that predicted angiographic restenosis on a segmental basis were 5.0 mm^2^ for the LCX ostium, 6.3 mm^2^ for the LAD ostium, 7.2 mm^2^ for the POC, and 8.2 mm^2^ for the proximal LM above the POC. Using these criteria, 133 (33.8 %) of the 403 patients had under-expansion of at least one of the pre-specified segments. In addition, under-expansion was more significantly frequent in the two-stent than in the single-stent group (54 % vs. 27 %, *p* = 0.001). In the two-stent group, the LCX ostium was the most common site of under-expansion (37 %), which may explain the greater risk of restenosis when distal LM bifurcation lesions are treated with a two-stent strategy. Overall, angiographic in-stent restenosis was significantly more frequent in lesions with than without under-expansion (24.1 % vs. 5.4 %, *p* = 0.001). Even in the two-stent group, the lesions with complete expansion at all sites showed a restenosis rate of only 6 %, similar to that in the single-stent group (6.3 %) or in patients with non-bifurcated LM coronary arteries (4.5 %). Furthermore, a smaller IVUS-MSA predicted angiographic in-stent restenosis 9 months after DES implantation to treat LM disease, and post-stenting under-expansion was an independent predictor of 2 year major adverse cardiac events, especially repeat revascularization. This study demonstrates that whichever 2-stent technique is chosen for the LM bifurcation disease, achieving sufficient post-stenting cross-sectional area is important for the favorable clinical outcomes.

Although the clinical impact of IVUS-guided stenting for unprotected LM coronary artery is unclear, as are several cost-benefit issues [38], this adjuvant method has been supported by several recent studies. For example, a subgroup analysis from the MAIN-COMPARE registry, including 201 propensity-score matched pairs, showed that 3 year mortality rates tended to be lower with IVUS than with angiography guidance (6.3 % vs. 13.6 %, log-rank *p* = 0.063; HR, 0.54; 95 % CI, 0.28–1.03) [14]. In particular, the 3 year mortality rates for the 145 matched pairs of patients undergoing DES implantation was significantly lower with IVUS than with angiography guidance (4.7 % vs. 16.0 %, log-rank *p* = 0.048; HR, 0.39; 95 % CI, 0.15–1.02). Interestingly, mortality rates started to diverge more than 1 year after the procedure. Since IVUS guidance did not reduce the risk of mortality in 47 matched pairs of patients undergoing bare metal stent implantation (8.6 % vs. 10.8 %, log-rank *p* = 0.35; HR, 0.59), this study indicated that IVUS guidance may play a role in reducing very late stent thrombosis and subsequent long-term mortality. A recent IVUS-TRONCO-ICP Spanish study also demonstrated the importance of IVUS surveillance during LM coronary artery stenting, with the incidence of the composite of cardiac death, myocardial infarction, and target lesion revascularization, as well as stent thrombosis at 3 years, being lower in the IVUS-guided group [15].

Frequency domain optical coherence tomography (OCT) is another attractive intravascular imaging tool for stent optimization. OCT offers superior resolution and can identify stent failure such as stent malopposition, edge dissections, tissue protrusion, and thrombus more clearly than IVUS [39]. Several pilot studies indeed showed that OCT-guided optimization of LM disease were feasible and safe [40, 41]. However, since blood must be adequately replaced by iodine contrast through a well-engaged guiding catheter for a clear image by OCT, evaluation of the LM ostium or a relatively large LM is often problematic. Furthermore, there is no standardized OCT criterion for optimizing stent implantation, particularly for the LM bifurcation, which hinders the use of this novel imaging modality to guide LM intervention. Nevertheless, with accumulating clinical data and experience, it is expected that OCT will be a useful adjunctive tool for the treatment of LM disease in near future.

## Conclusions

**Fig. 3:**
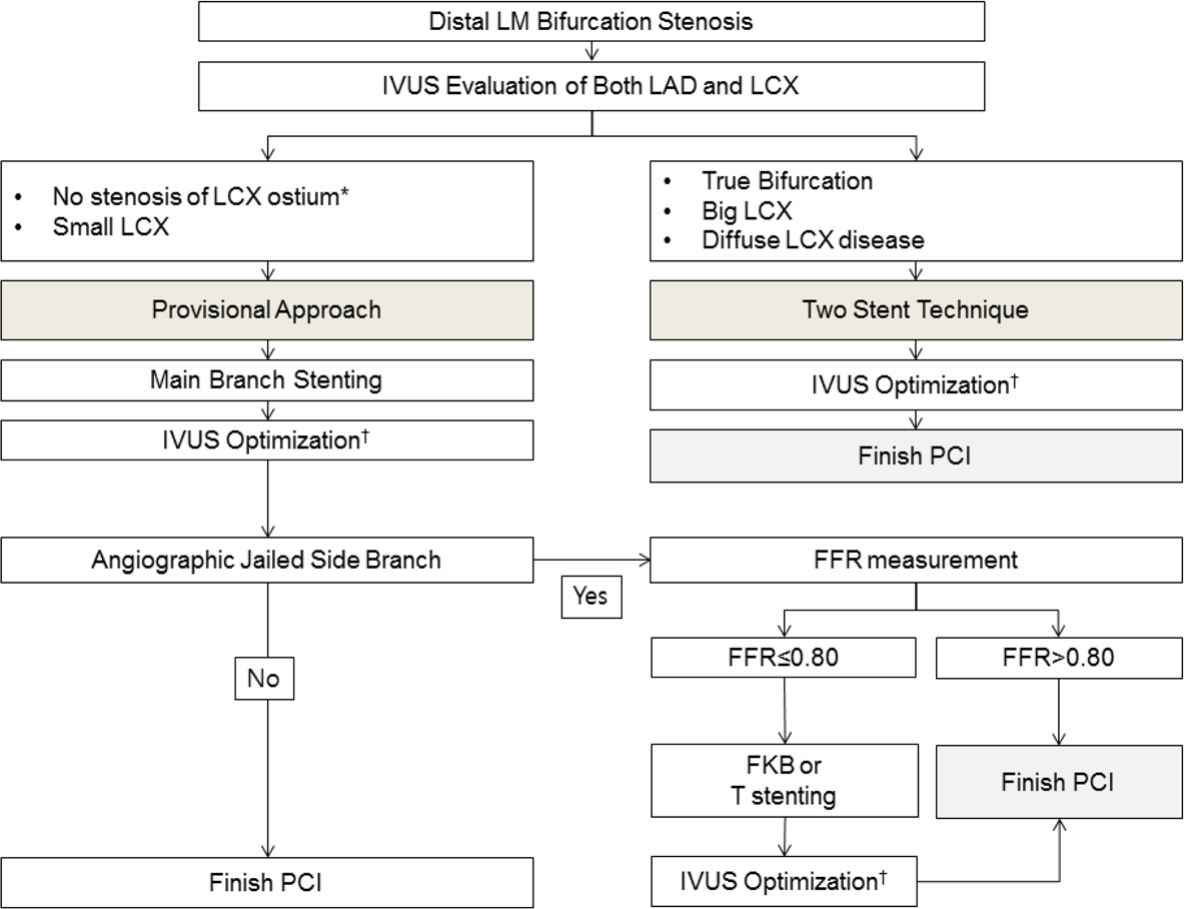
Flow chart for the interventional treatment of distal left main bifurcation lesions. *In general, minimal lumen area >4 mm2 or plaque burden <50 % of the ostium of the left circumflex artery is considered insignificant stenosis. †The stent should be well opposed to the vessel wall and sufficiently expanded to avoid restenosis (minimal stent area: 5 mm2 for the ostium of the left circumflex artery, 6 mm2 for the proximal left anterior descending artery, 7 mm2 for the polygon of confluence, and 8 mm2 for the distal left main artery), without procedure-related complications. Abbreviations: FKB, final kissing balloon; IVUS, intravascular ultrasound; LAD, left anterior descending artery; LCX, left circumflex artery; LM, left main; PCI, percutaneous coronary intervention

Figure 3 is a schematic flow chart summarizing clinical strategies for treating distal LM bifurcation disease. Careful selection of candidates for the provisional approach is the most important step in avoiding procedure-related complications and ensuring favorable individual outcomes. Incorporating the FFR-guided PCI strategy in treating isolated LCX may further help avoid unnecessary SB interventions. Meticulous evaluation of LM bifurcations using intravascular imaging is crucial in selecting the proper stent strategy and in achieving optimal stent results.

## Abbreviations

CI: confidence interval
DES: drug-eluting stent
FFR: functional flow reserve
FKI: final kissing balloon inflation
HR: hazard ratio
IVUS: intravascular ultrasound
LCX: left circumflex artery
LM: left main
MB: main branch
MSA: minimal stent area
MV: main vessel
OCT: optical coherence tomography
PCI: percutaneous coronary intervention
POC: polygon of confluence
SB: side branch
